# Co-inhibition of CD73 and ADORA2B Improves Long-Term Cigarette Smoke Induced Lung Injury

**DOI:** 10.3389/fphys.2021.614330

**Published:** 2021-01-28

**Authors:** Zhi Tian, Jendayi Dixon, Xiaofang Guo, Benjamin Deal, Qianjin Liao, Yujuan Zhou, Feng Cheng, Diane S. Allen-Gipson

**Affiliations:** ^1^Department of Pharmaceutical Sciences, Taneja College of Pharmacy, University of South Florida, Tampa, FL, United States; ^2^Department of Obstetrics and Gynecology, Morsani College of Medicine, University of South Florida, Tampa, FL, United States; ^3^Hunan Cancer Hospital and The Affiliated Cancer Hospital of Xiangya School of Medicine, Central South University, Changsha, China; ^4^Division of Allergy and Immunology, Department of Internal Medicine, Morsani College of Medicine, University of South Florida, Tampa, FL, United States

**Keywords:** CD73, adenosine, cigarette smoke, ECIS, lung

## Abstract

Adenosine (ADO) involvement in lung injury depends on the activation of its receptors. The ADO A_2A_ receptor (ADORA2A) and A_2B_ receptor (ADORA2B) are best described to have both tissue-protective and tissue-destructive processes. However, no approach has been effective in delineating the mechanism(s) involved with ADO shifting from its tissue-protective to tissue-destructive properties in chronic airway injury. Using cigarette smoke (CS) as our model of injury, we chronically exposed Nuli-1 cells to 5% CS extract (CSE) for 3 years establishing a long-term CSE exposure model (LTC). We found significant morphological changes, decreased proliferation, and migration resulting in impaired airway wound closure in LTC. Further investigations showed that long-term CSE exposure upregulates CD73 and ADORA2B expression, increases ADO production, inhibits PKC alpha activity and p-ERK signaling pathway. Knocking down ADORA2B and/or CD73 in LTC activates PKC alpha and increases p-ERK signaling. Knocking down both showed better improvement in wound repair than either alone. *In vivo* experiments also showed that double knockout CD73 and ADORA2B remarkably improved CS-induced lung injury by activating PKC alpha, reducing the inflammatory cell number in bronchoalveolar lavage fluid and the production of inflammatory mediator IL-6, inhibiting the fibrosis-like lesions and decreasing collagen deposition surrounding bronchioles. Collectively, long-term CSE exposure upregulates CD73 expression and increases ADO production, which promotes low affinity ADORA2B activation and subsequent diminution of PKC alpha activity and ERK signaling pathway, and inhibition of airway wound repair. Moreover, the data suggesting ADORA2B and CD73 as potential therapeutic targets may be more efficacious in improving chronic CS lung diseases and impaired wound repair.

## Introduction

Cigarette smoke (CS) is a major risk factor for several chronic lung diseases including asthma, emphysema, and chronic obstructive pulmonary disease (COPD). COPD is a serious burden throughout the word both economically and socially and is characterized by chronic inflammation and injury of both the airways and the parenchymal structures of the lung. It was recognized as the third leading causes of mortality and morbidity in the United States. An estimated 95% of COPD cases are caused by CS, not only smokers but also those involuntary exposed to second hand smoke (Barnes et al., 2003).

Adenosine (ADO) is a purinergic molecule which modulates tissue damage and repair (Fredholm, 2007). It is best known for promoting anti-inflammatory activities during acute injury, whereas elevations in ADO contribute to destructive tissue remodeling processes in chronic injury (Hasko and Pacher, 2008; Zhou et al., 2009). ADO levels are elevated in the lungs of patients with COPD, where it is believed that the balance between tissue repair and excessive airway remodeling is regulated through its receptors (A_1_, A_2A_, A_2B_, and A_3_) (Zhou et al., 2010). Recent studies have identified the ADORA2B as an important target in the regulation of both acute and chronic lung disease with opposing activities. Several studies have demonstrated a protective role of A_2B_ adenosine receptor (AR) during acute lung injuries (Ahmad et al., 2009; Eckle et al., 2009; Konrad et al., 2017). However, there is a substantial amount of evidence indicating that ADORA2B also has a non-protective role; for instance, smokers with COPD have elevated mRNA levels of ADORA2B as compared to non-smokers and smokers without COPD (Sun et al., 2006; Zhou et al., 2009, 2010; Karmouty-Quintana et al., 2013). No approach has been effective in delineating the mechanism(s) involved with ADO shifting from its tissue-protective to tissue-destructive properties, which may implicate the ADORA2B in contributing the tissue destructive events observed in COPD.

CS can induce release of ATP both *in vitro* and *in vivo* (Lommatzsch et al., 2010); ATP degradation is regulated by ectonucleoside triphosphate disphosphohydrolase (CD39) that hydrolyzes ATP and ADP to AMP and subsequently to ADO by CD73, an ecto-5′nucleotidase (also known as NT5E) that is found in most tissues (Thome et al., 2009). CD73-generated extracellular ADO is known to regulate all four ARs (Burnstock, 2008); however, high concentration levels (in the micromole range) of endogenous ADO is required to activate the low affinity receptor, ADORA2B (Beukers et al., 2000; Hamann et al., 2015). CD73 has been reported that it also has both protective and promoting effects in lung inflammation and fibrosis (Volmer et al., 2006; Ehrentraut et al., 2013; Bou Ghanem et al., 2015; Wirsdorfer et al., 2016; Minor et al., 2019). In a bleomycin induced lung injury model, CD73 knock out (KO) mice exhibited enhanced inflammation and collagen production (Volmer et al., 2006). However, the radiation-induced lung epithelial damage and fibrosis was significantly blunted in CD73 KO mice (Wirsdorfer et al., 2016). There are no studies correlating the effect of CS on the activities of CD73 and subsequent purinergic signaling pathway in airway epithelial cells that may play a role in shifting the tissue protective to tissue destructive properties in ADO-mediated wound repair. We proposed to investigate CD73 role in switching on the adenosinergic signaling by catalyzing the hydrolysis of AMP into ADO, subsequently up-regulating ADORA2B.

## Materials and Methods

### Cell Culture

The Nuli-1 human bronchial epithelial cell line was obtained from the American Type Culture Collection (Rockville, MD). Cells were cultured on type VI placenta collagen (Sigma, St. Louis, MA) pre-coated dishes in bronchial epithelial growth media (BEGM; Lonza, Walkersville MD). Cells were maintained in humidified incubator at 37°C in an atmosphere of 5% CO_2_ as described previously (Tian et al., 2017). Cell images were taken by EVOS XL imaging system (Life technologies, United States).

### Animals

C57BL/6 background CD73 KO mice were purchased from the Jackson Laboratories (Bar Harbor, ME). C57BL/6 background ADORA2B KO mice were a gift from Dr. Michael Blackburn (University of Texas Medical School at Houston, McGovern Medical School, Houston TX). WT and ADORA2B/CD73 double KO (DKO) mice were generated by cross breeding ADORA2B KO and CD73 KO mice. Genotyping was performed by Transnetyx^®^ (Transnetyx, Cordova, TN) using real-time PCR. All mice used for experiments were between 8 and 10 weeks and were maintained under standard housing conditions in the animal care facility at the University of South Florida (USF). All studies were carried out in accordance with the *Guide for the Care and Use of Laboratory Animals of the National Institutes of Health* and were approved by USF Institutional Animal Care and Use Committees.

### Generation of Long-Term CS Extract (CSE) Exposed Nuli-1 Cells (LTC-Nuli)

**FIGURE 1:**
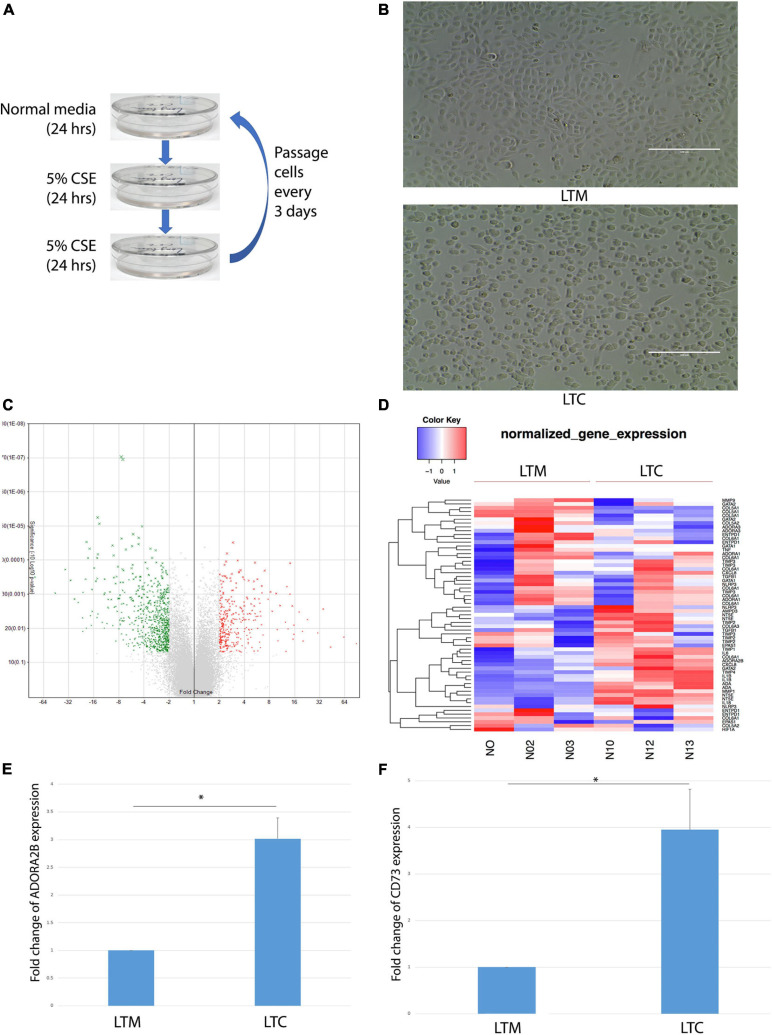
**(A)** Workflow of long-term CSE exposure. **(B)** Morphological changes observed in LTC. Scale bar, 100 μm. **(C)** Volcano plots showed that over 1,000 genes changed at least twofold after long-term CSE exposure. Red indicates up-regulated and green indicates down-regulated genes. **(D)** Heat map of up- or down- regulated wound repair related genes that are differentially expressed (> twofold) in LTC. Red indicates up-regulated and blue indicates down-regulated genes as shown in the scale bar. **(E)** ADORA2B expression level up-regulated after long-term CSE exposure * indicated *p* < 0.05. **(F)** CD73 expression level up-regulated after long-term CSE exposure * indicated *p* < 0.05.

CSE was prepared as previously described using 3R4F reference cigarettes (University of Kentucky, Lexington, KY) (Tian et al., 2017). Nuli-1 cells were treated 1 day in normal media, and then 2 days in media containing 5% CSE; cells (designated LTC) were passaged every 3 days as shown in Figure 1A. Control Nuli-1 cell (designated LTM) culture in normal media and passaged at same time as LTC. Both LTM and LTC are cultured for more than 3 years.

### Microarray Analysis

RNA was extracted using TRIzol^®^ reagent (Invitrogen, Carlsbad, CA) according to the manufacturer’s instructions. The Molecular Genomics Core at the H. Lee Moffitt Cancer Center and Research Institute performed the Affymetrix arrays. The data has been uploaded to NCBI, the GEO submission number is GSE111952.

### PKC Alpha Activity Assay

PKC activity from Nuli-1 cell was determined using PKC alpha kinase enzyme system (Promega, Madison, WI) according to the manufacturer’s instructions. Briefly, cells lysates were prepared in RIPA buffer (Cell signaling, Danvers, MA). Equal amounts of protein lysates (5 μl) were co-incubated with PKC alpha mixture containing 1 μl of CREBtide, 2.5 μl of 10× PKC lipid activator and 0.125 μl of 200× ATP in 96 well plate at room temperature (RT) for 60 min. At the end of incubation, 5 μl of ADP-Glo reagent was added and incubated at RT 40 min followed by 10 μl of kinase detection reagent for a final incubation at RT for 60 min. PKC alpha was measured via luminescence signal using the BioTek H1 plate reader (BioTek Inc., Winooski, Vermont). PKC activity from mice trachea was determined in crude whole-cell fractions of bronchial epithelial cells, using a modification of procedures previously described (Jiang et al., 1992; Allen-Gipson et al., 2013).

### Cell Proliferation Assay

8W10E + ECIS culturewares (Applied BioPhysics, Troy, NY) were used for proliferation assay as described before (Tian et al., 2017). Briefly, 8 × 10^3^ cells were seeded per well and cultured in incubator at 37°C in an atmosphere of 5% CO_2_. Media were changed next day and the resistances were recorded in real-time at 4,000 Hz using ECIS Zθ instrument (Applied BioPhysics, Troy, NY).

### Cell Migration Assay

8W1E ECIS culturewares (Applied BioPhysics, Troy, NY) were used for migration assay as described previously (Tian et al., 2017). Briefly, 8 × 10^4^ cells were seeded each well and cultured in incubator at 37°C in an atmosphere of 5% CO_2_. Media were changed next day; and cell monolayer were wounded using an elevated field pulse of 3,000 μA at 80,000 Hz applied for 20 s, producing a uniform circular lesion 250 μm in size. The wounds were tracked in real-time at 4,000 Hz using ECIS Zθ instrument (Applied BioPhysics, Troy, NY).

### Cell Barrier Function Assay

8W10E + culturewares were used for barrier function assay, 8 × 10^4^ cells were seeded in each well and the resistance was measured immediately at 2,000 Hz and 64,000 Hz using ECIS Zθ instrument (Applied BioPhysics, Troy, NY).

### Taqman Real-Time PCR

RNA was extracted using TRIzol^®^ reagent (Invitrogen, United States) according to the manufacturer’s instructions. cDNA was synthesized by using 100 ng of total RNA and TaqMan reverse transcription kit (Applied Biosystems, Foster City, CA). Real-time PCR reactions were prepared in triplicate using 1× TaqMan Master Mix (Applied Biosystems, Foster City, CA) and primers and probes, as previously described (Tian et al., 2017). TaqMan real-time PCR was performed using an ABI 7,900 Sequence Detection System (Applied Biosystems, Foster City, CA). The relative fold change was calculated by the 2^−ΔΔCt^ method.

### Knockdown of Gene Expression by shRNA

MISSION shRNA Lentiviral transduction particles were purchased from Sigma-Aldrich (St. Louis, MO). 2 × 10^4^ LTC cells were plated in a 96-well plate and incubated overnight (ON). Fresh experimental media were added containing 110 μl 0.8 μg Hexadimethrine bromide (Sigma, St. Louis, MO) and 10 μl of lentiviral shRNA particles to each well and incubated ON. Following the transduction, experimental media were replaced with normal media ON, and puromycin (10 μg/ml) was added to remove any non-transduced cells.

### Western Blot

Cells lysates were prepared in RIPA buffer (Cell signaling, Danvers, MA) containing PMSF (0.5 mM). Equal amounts of protein lysates were separated by 10% sodium dodecyl sulfate-polyacrylamide gel electrophoresis (SDS-PAGE) and transferred on polyvinylidene difluoride (PVDF) membranes. Membranes were blocked at RT for 1 h with 5% bovine serum albumin (BSA, Thermo Fisher Scientific, Hampton, NH) followed by exposure to different primary antibody, including anti-ADORA2B (1:200, Abcam, Cambridge, United Kingdom), anti-CD73 (1:1,000, Cell signaling, Danvers, MA), anti-ZO-1 (1:1,000, Cell signaling, Danvers, MA), anti-p-MEK (1:1,000, Cell signaling, Danvers, MA), anti-p-ERK (1:1,000, Cell signaling Danvers MA), anti-p-p90RSK (1:1,000, Cell signaling, Danvers, MA), anti-p-CREB (1:1,000, Cell signaling, Danvers, MA), anti-GAPDH (1:2,000, Cell signaling, Danvers, MA), ON. After washing with Tris-Buffered Saline (TBS) plus 1% Tween-20, membranes were incubated with 1:5,000 diluted goat anti rabbit IgG-HRP secondary antibody for 1 h at RT. Pierce^TM^ Western Blotting Substrate (Thermo Fisher Scientific, Hampton, NH) was used and membranes were imaged via Bio-red Doc. All the WB image we presented in this manuscript is representative of 3 experiments conducted on three separate occasion. Antibody to GAPDH (Cell signaling, Danvers, MA) was used as a loading control. Densitometric analysis of Western blots were analyzed by ImageJ (Public domain, BSD-2, NIH, United States).

### High-Performance Liquid Chromatography (HPLC) Measurement of Extracellular ADO

ADO was determined using HPLC as described with modifications (Fu et al., 2000). Briefly, 1 ml of cell culture supernatant was mixed with 50 μl of perchloric acid for 5 min, followed by centrifugation at 8,000 g for 5 min. The supernatant was transferred to a micro-insert tube (placed in a brown vial) for analysis using Agilent 1,260 infinity HPLC system (Agilent Technologier, Santa Clara, CA) with Luna 5 u C18(2) 100 A column (Phenomenex, United States) guarded by a SecurityGuard^TM^ Cartridge System (Torrance, CA). The injection volume was 100 μl and total running time was 20 min. The mobile phase was prepared as follows: 50 mM of sodium perchlorate, 0.1 M of sodium acetate, 2.4 mM of sodium 1-heptanesulfonate, 0.9% acetonitrile and 0.1 M of sodium azide. The flow rate was 2 ml/min and the ADO retention time was 7.7 min.

### CD73 Activity Assay

Enzymatic activities for CD73 in Nuli-1 cells were performed using CD73 Activity Assay Kit (Abcam, Cambridge, United Kingdom) according to the manufacturer’s instructions. Briefly, 1 × 10^6^ cells were prepared in 100 μl of Assay Buffer for 10 min at 4°C. The supernatant was collected by centrifuging at 10,000 × g for 10 min at 4°C and 100 μg of the protein sample was added into a 96 well-plate. The sample then incubated with CD73 Reaction Mix at 37°C for 20 min. The incubation reaction stopped by adding 4 μl of Stop Solution and followed by 80 μl of CD73 Developer I and 40 μl of Developer II. Each well incubated at RT for 20 min and the absorbance were recorded at 670 nm using SPECTRA MAX 190 (Analytical instrument brokers, LLC, Golden Valley, MN).

### CS and Air Exposure

A total of 10 mice (5 males and 5 females) in each group (WT, ADORA2B KO, CD73 KO, and ADORA2B/CD73 DKO) were passively treated with CS using a Teague-10 Smoking Machine (Teague Enterprises, Davis, CA). Using the Teague device, mice were exposed to smoke from eighty 3R4F reference cigarettes (University of Kentucky, Lexington, KY) per day. Mice receiving CS were gradually brought to their target exposure over a period of 5 days, treated 5 days/week for 3 months. The same amount of control mice from each group were exposed to air in the same manner in a similar apparatus for the same periods of time.

### Bronchoalveolar Lavage Fluid (BALF)

Mice were euthanized with a cocktail of xylazine and ketamine (0.1 mL/10 g). BALF were collected as previously described (Tian et al., 2017). Tracheas were exposed and the ends of the tracheas were tied off, a total of 1.0 mL cold sterile PBS (Gibco, Grand Island, NY) was gently flushed into the lungs and recovered. Collected BALF was centrifuged at 300 g for 7 min at 4°C. Pelleted cells were resuspended in 1.0 ml of PBS. Total cells were counted on a hemocytometer, and 1–5 × 10^3^ cells were spun onto glass microscope slides (cytospin 3; Shandon Scientific, Cheshire, United Kingdom). Cells were air dried for 24–36 h, fixed, and stained with a HEMA 3 stain set (Thermo Fisher Scientific, Kalamazoo, MI). Differential cell counts of at least 300 cells per slide were made according to morphological criteria. The number of cells recovered was calculated and expressed as absolute cell numbers.

### Lung Collection, Histology, and Collagen Staining

Whole lungs were excised and inflated to 10 cm H_2_O pressure with 10% formalin (Sigma, St. Louis, MO) to preserve pulmonary architecture. Lungs were embedded in paraffin, and sections were cut (5 μm) and processed for hematoxylin and eosin staining. Rehydrated lung sections were stained with Picro-Sirius Red Solution (Abcam, Cambridge, United Kingdom) to determine bronchial airway collagen deposition according to the manufacturer’s instructions. Briefly, the rehydrated lung sections were incubated with Picro-Sirius Red Solution for 60 min at RT, then rinsed twice by 0.5% Acetic Acid Solution (Thermo Fisher Scientific, Rockford, IL) and once by absolute alcohol (Sigma, St. Louis, MO). The slides were cleared and dehydrated in absolute alcohol, mounted with mounting medium (Thermo Fisher Scientific, Rockford, IL) and cover slips. The images were taken by EVOS XL imaging system (Life technologies, United States). For quantitative histology, airways were grouped by size in diameter of 100–200 μm, the percentage of collagen in each image were measured using ImageJ (NIH, United States).

### Statistics Analysis

All experiments were conducted in triplicate and results were expressed as mean ± SE. Data were statistically analyzed using Student’s paired *t*-test followed by Tukey’s multiple-comparison test. Statistical differences among groups were determined using one-way ANOVA followed by Tukey’s multiple-comparison test. Significance was assigned at P < 0.05.

## Results

### Microarray Analysis of Gene Expression Profile in Long-Term CSE Exposed Airway Epithelial Cell

To assess the long-term exposure of CSE, Nuli-1 cells were treated in cycles of 24 h in 100% media followed by 48 h of 5% CSE treatment (1 cycle); repeated over the course of 3 years (LTC); the media control Nuli-1 cells were exposed long-term with 100% normal media (LTM) as shown in Figure 1A. After 3 years of CSE exposure, LTC showed visually appreciable morphological changes when compared to LTM. LTC took on a more rounded shape with less physical contact with each other (Figure 1B). Affymetrix array revealed at least twofold changes in over 1,000 genes in LTC (Figure 1C). The heatmap in Figure 1D showed that CD73 (NT5E) and ADORA2B were upregulated by CSE exposure (Figure 1D). Moreover, the ADORA2B gene revealed a threefold increase in expression (Figure 1E) and the CD73 was upregulated by 3.94-fold (Figure 1F). Collectively, data revealed the long-term CSE promote physical and gene changes in the Nuli-1, which may contribute to the CS-dysregulation of airway-wound repair.

### Long-Term CSE Exposure Impairs Airway Wound Repair

**FIGURE 2:**
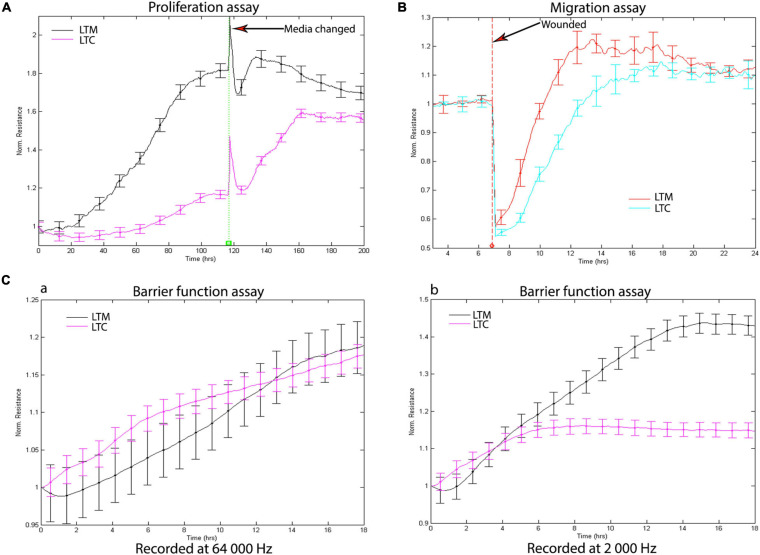
**(A)** Long-term CSE exposure inhibits Nuli-1 cells proliferation. Media were changed after 118 h. Signals were recorded at 4,000 Hz. Each condition was conducted in triplicate. Statistically differences observed after 24 h (*P* < 0.05) **(B)** Long-term CSE exposure inhibits Nuli-1 cells migration. The resistances were measured at 4,000 Hz. Each condition was conducted in triplicate. Statistically differences observed after 7.5 h (*P* < 0.05) **(C)** Long-term CSE exposure impairs Nuli-1 cells barrier function. a. Resistances were recorded at 64 000 Hz; b. Resistances were recorded at 2,000 Hz. The resistances were measured simultaneously. Each condition was conducted in triplicate. Statistically differences observed after 7 h (*P* < 0.05).

The proliferation, migration, and barrier function are regarded as important repair processes in wound repair; the ECIS was used to assess how long term CSE exposure affects these processes. Consistent with the morphological changes, long-term CSE exposure not only decreased the rate of proliferation (Figure 2A), but also reduced the rate of migration (Figure 2B). Additionally, we measured the barrier function of the CSE exposed cells using ECIS barrier function assay. Electric current can couple capacitively through cells membranes in high AC frequencies (e.g., 64,000 Hz), while moving through the paracellular passage between the cells (the barrier function) in low AC frequencies (e.g., 2,000 Hz). With the cell attached to the ECIS culture-well, the resistance will increase consistently. The measured resistance at 64,000 Hz were similar, which suggests there were the same number of cells attached to the ECIS culture-well (Figure 2Ca); however, the recorded resistance at 2,000 Hz from LTC were decreased when compared to LTM (Figure 2Cb), indicating that CSE treatment blunted barrier function of LTC. Collectively, the data indicate long-term CSE treatment impaired critical processes necessary for airway wound repair.

### Long-Term CSE Exposure Triggers ADORA2B-Mediated Tissue Destructive Sensors Through Inhibition of p-ERK Pathway

**FIGURE 3:**
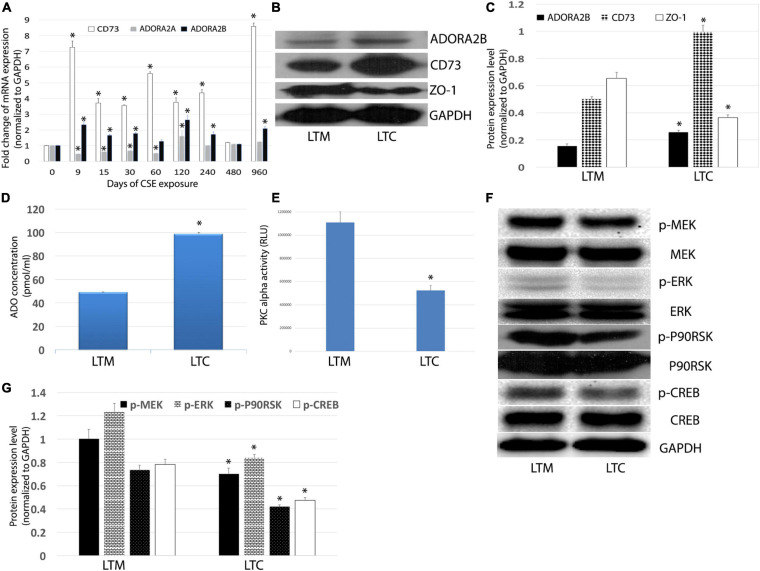
**(A)** Effects of longterm CSE on transcript levels of ADORA2B. Expression of mRNA in airway epithelial cells treated with CSE for 3 years. Values are mean ± SE, ^∗^ indicated *p* < 0.05. **(B)** CSE stimulates ADORA2B, CD73, and ZO-1 protein expression. **(C)** Normalized densitometry analysis of ADORA2B, CD73, and ZO-1. ^∗^ indicated *p* < 0.05. **(D)** Extracellular ADO regulated by long-term CSE exposure. The extracellular ADO concentration was measured using HPLC. Data are mean ± SE, ^∗^ indicated *p* < 0.05. **(E)** Activation of PKC alpha inhibited by long-term CSE exposure. Data are mean ± SE, ^∗^ indicated *p* < 0.05. **(F)** Long-term CSE exposure inhibits phosphorylation of MEK, ERK, p90RSK, and CREB. **(G)** Normalized densitometry analysis of p-MEK, p-ERK, p-p90RSK, and p-CREB. Data are mean ± SE, ^∗^ indicated *p* < 0.05.

As the low-affinity adenosine receptor, the ADORA2B has been implicated in tissue injury as it relates to COPD. Most recently we reported significant increase in transcriptional expression of ADORA2B in CS-treated mice (Tian et al., 2017), however, very little is known regarding long-term CSE exposure on ADORA2B tissue destructive signaling. To track the change of CD73, ADORA2A, and ADORA2B, we analyzed the mRNA expression level in the different time point of exposure. The ADORA2A expression decreased in the early stage of CSE exposure, but it back to normal after 8-month exposure. The expression level of both CD73 and ADORA2B were upregulated (Figure 3A). Western Blot also confirmed an increase in the ADORA2B expression as well as CD73 expression (Figures 3B,C), which may contribute to the increase in extracellular ADO concentration observed in LTC (Figure 3D).

To further understand the mechanisms of ADORA2B-mediated tissue destructive properties, we investigated the involvement of PKC activity and downstream MAPKs signaling pathway. Interestingly, we observed a significant decrease in the tight junction protein ZO-1 in LTC (Figures 3B,C), which infers the observed reduced barrier function (Figure 2C) may also be contributed to decreased ZO-1. Likewise, luminescence assay revealed that long-term CSE exposure significantly diminished PKC alpha activity in Nuli-1 cells (Figure 3E). Moreover, the phosphorylation of ERK/MEK, as well as downstream p90RSK and CREB in LTC were decreased (Figures 3F,G), but there were no significant changes observed in phosphorylation of JNK or p38MAPK (data not shown), which indicates long-term CSE may regulate airway wound repair via inhibiting p-ERK pathway.

### Knockdown of ADORA2B and CD73 or Double Knockdown Ameliorates Long-Term CSE Impaired Airway Wound Repair

**FIGURE 4:**
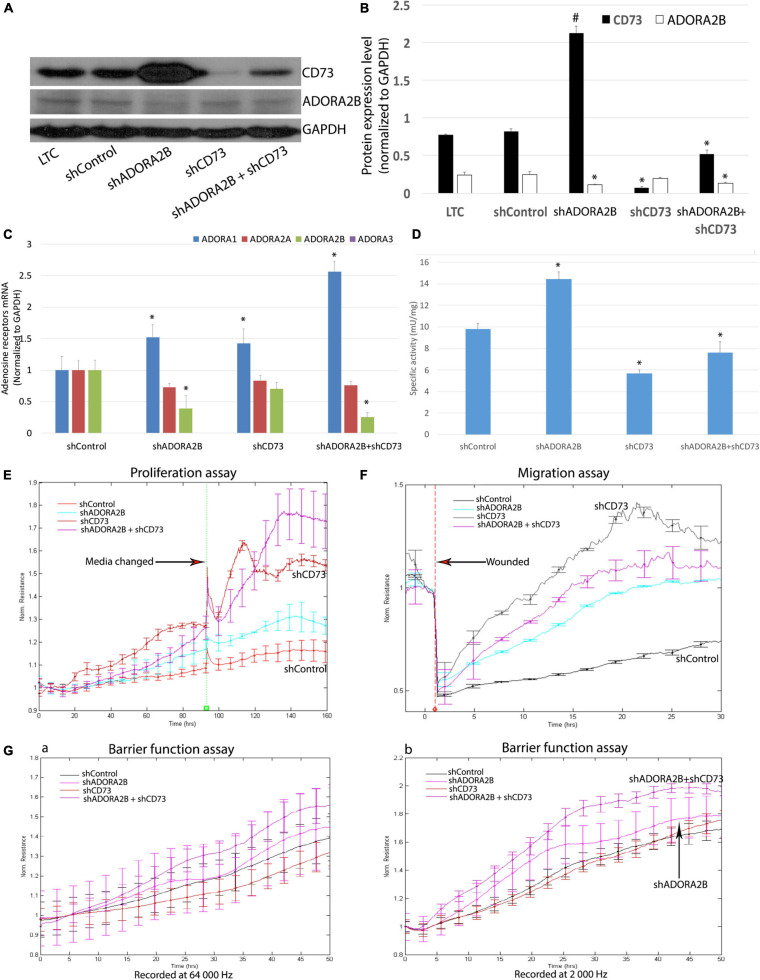
**(A)** Efficient ADORA2B and/or CD73 knock down in LTC. **(B)** Normalized densitometry analysis of ADORA2B and CD73. Values are mean ± SE, ^∗^ indicated *p* < 0.05, # indicated that CD73 protein expression level significantly increased, *p* < 0.05. **(C)** Transcript levels of ADORA1, ADORA2A, ADORA2B, and ADORA3 in Nuli-1 cells. ^∗^ indicates significance value *P* < 0.05 compared with shControl group. The transcript level of ADORA3 was not detected in Nuli-1 cells. **(D)** CD73 enzyme activity in Nuli-1 cells. ^∗^ indicates significance value *P* < 0.05 compared with shControl group. **(E)** Targeting both ADORA2B and CD73 ameliorates long-term CSE impaired cell proliferation. Media were changed after 96 h. Signals were recorded at 4,000 Hz. Each condition was conducted in triplicate. Statistically differences observed after 80 h (*P* < 0.05). **(F)** Targeting both ADORA2B and CD73 ameliorates long-term CSE impaired cell migration. The resistances were measured at 4,000 Hz. Each condition was conducted in triplicate. Statistically differences observed after 6 h (*P* < 0.05). **(G)** Targeting both ADORA2B and CD73 ameliorates long-term CSE impaired cell barrier function. a. Resistances were recorded at 64,000 Hz; b. Resistances were recorded at 2,000 Hz. The resistances were measured simultaneously. Each condition was conducted in triplicate. Statistically differences observed after 27 h (*P* < 0.05).

Our microarray analysis revealed increased gene expression of CD73 and ADORA2B. To determine whether limiting the generation of adenosine and adenosine signaling can affect long-term CSE-impaired airway wound repair, we knocked down CD73 and/or ADORA2B in LTC. We observed when ADORA2B was knocked down, there was a significant increase in CD73 expression (Figures 4A–C). Consistent with the changes of CD73 protein and mRNA expression level in each group, the enzyme activities of CD73 also changed at the same manner (Figure 4D). Proliferation (Figure 4E) and migration (Figure 4F) studies also revealed knocking down either CD73 or ADORA2B increased the rate of proliferation and migration while knocking down both CD73 and ADORA2B demonstrated significant improvement than individually knocking down each gene. Furthermore, when we double knocked-down CD73 and ADORA2B, this group showed improvement of barrier function; however, there were no significant changes observed in either shCD73 or shADORA2B cells (Figure 4G). Consequently, our data suggest knocking down in combination CD73 and ADORA2B as potential therapeutic strategy to ameliorate long-term CSE impaired airway wound repair.

### Targeting CD73 and ADORA2B Improves Chronic CSE Impaired Wound Repair via Activating p-ERK Signaling Pathway

**FIGURE 5:**
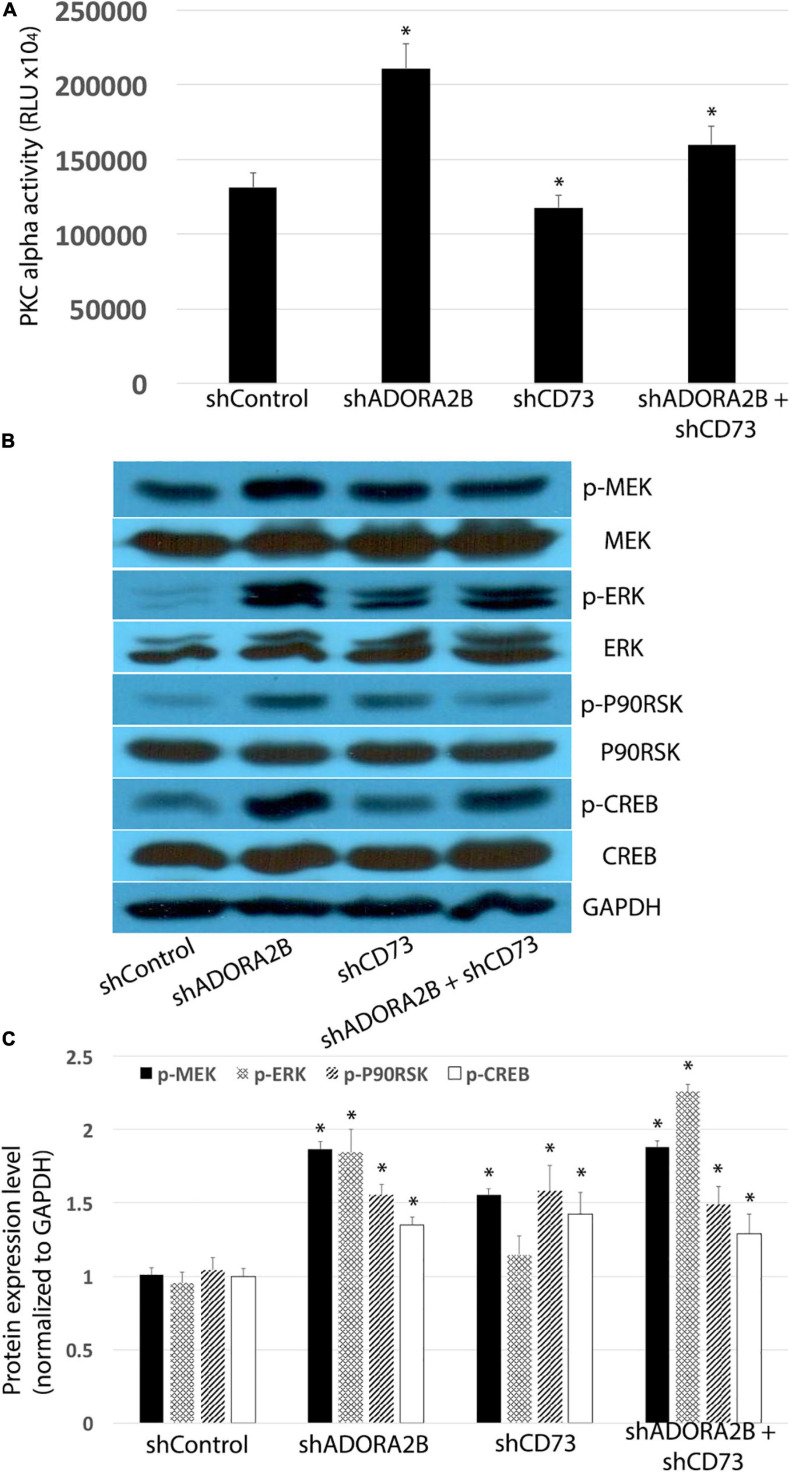
**(A)** Knocking down ADORA2B alone or both ADORA2B and CD73 activates PKC alpha in LTC. Data are mean ± SE, ^∗^ indicated *p* < 0.05. **(B)** Knocking down ADORA2B and CD73 activates MEK/ERK/p90RSK/CREB signaling pathway. **(C)** Normalized densitometry analysis of p-MEK, p-ERK, p-p90RSK, and p-CREB. Data are mean ± SE, ^∗^ indicated *p* < 0.05.

Unlike the acute CSE exposure, long-term CSE exposure inhibited PKC alpha activity in airway epithelial cells and subsequently decreased phosphorylation of ERK. Both shADORA2B and/or shADORA2B + shCD73 combined groups significantly increased PKC alpha activity; however, there were no significant changes observed in shCD73 group alone (Figure 5A). Furthermore, knocking down CD73 and/or ADORA2B significantly increased phosphorylation of MEK and ERK, as well as downstream p90RSK and CREB (Figures 5B,C). Collectively, our data suggest long-term CSE activation of CD73 and ADORA2B affect down-stream events that are critical to airway wound repair.

### Double Knockout of ADORA2B and CD73 Impairs Chronic CS Induced Lung Injury in Mice

**FIGURE 6:**
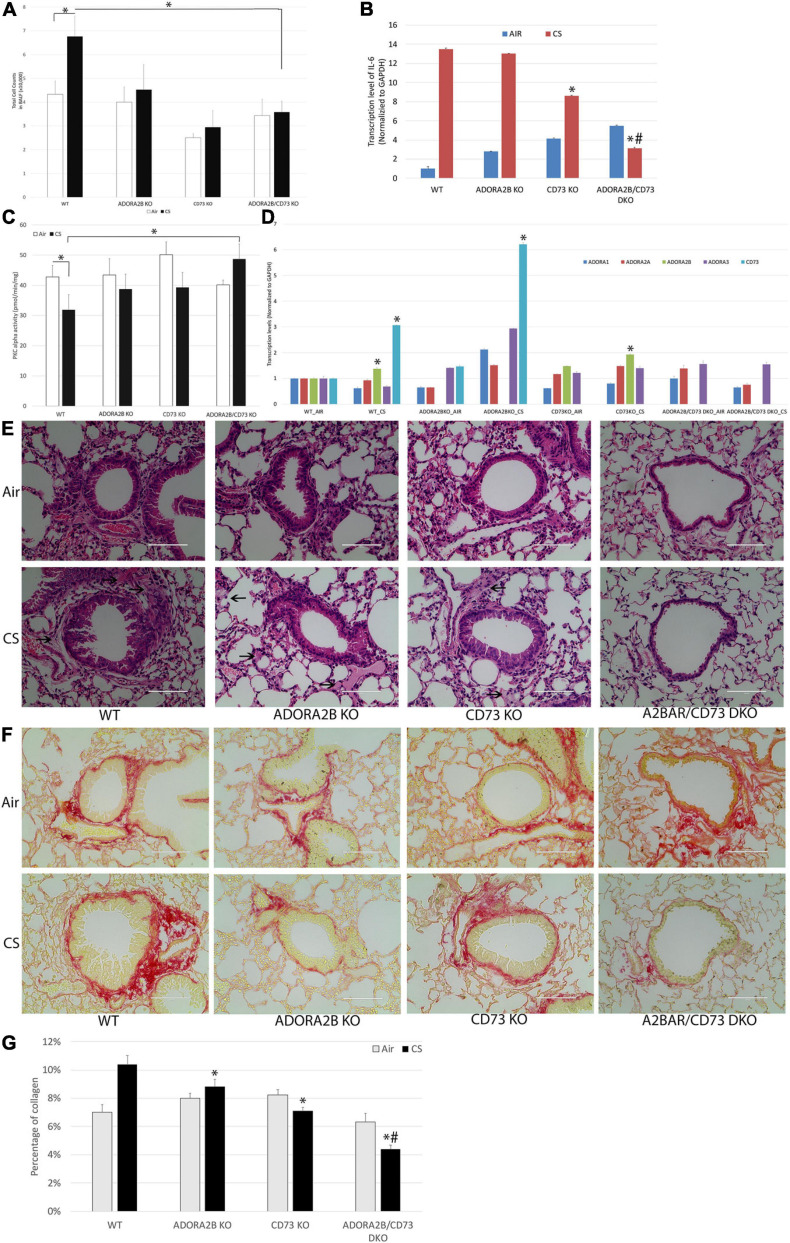
Double knockout CD73 and ADORA2B improves CS-induced lung injury. **(A)** CD73/ADORA2B DKO decreases CS-induced inflammatory cells in BALF. Data are mean ± SE, ^∗^ indicated *p* < 0.05. *n* = 6. **(B)** Transcript levels of IL-6 in mice. ^∗^ indicates significance value *P* < 0.05 compared with WT mice. # indicates significance value *P* < 0.05 compared with ADORA2B KO and CD73 KO mice. **(C)** CD73/ADORA2B DKO activates chronic CS treatment inhibited PKC alpha. Data are mean ± SE, ^∗^ indicated *p* < 0.05. *n* = 6. **(D)** Transcript levels of ADORA1, ADORA2A, ADORA2B, ADORA3, and CD73 in mice. ^∗^ indicates significance value *P* < 0.05 compared with their AIR control group. **(E)** Photomicrographs of representative histopathological profiles. Sections of mouse lung tissue were paraffin-embedded and stained with hematoxylin and eosin in each cohort. Arrows show fibrosis-like changes comprised of connective tissue and extracellular matrix present in samples. Scale bar, 100 μm. **(F)** Representative micrograph of lung sections from different groups of mice. Sections were stained with Picro-Sirius Red Solution (collagen was highlighted as red color, muscle fibers and cytoplasm were highlighted as yellow). Scale bar, 100 μm. **(G)** Quantitative analysis of lung collagen contents by ImageJ were expressed as Mean ± SD. ^∗^ indicated *p* < 0.05 compared with WT-CS group. # indicated *p* < 0.05 compared with ADORA2B KO and CD73 KO mice. More abundant collagen is accumulated in CS-treated WT mice as compared to WT control mice, and double knockout ADORA2B and CD73 dramatically reduced collagen accumulation.

We studied the effects of CS as a model of airway damage to elucidate the mechanisms of co-inhibition of CD73 and ADORA2B improves wound repair in mice exposed to CS for 3 months. WT mice treated with CS increased the total number of BAL cells compared to WT control animals; however, there is a significant decrease in the knockout CD73 and/or ADORA2B mice compared to the CS-treated WT mice (Figure 6A). The expression level of pro-inflammatory cytokine interleukin-6 (IL-6) also significantly increased in CS group, and remarkably decreased in CD73/ADORA2B DKO mice while compared with control mice (Figure 6B). Consistent with our *in vitro* results, 3 months CS exposure decreased the activity of PKCα in WT mice. Interestingly, CD73/ADORA2B DKO mice have the same activity of PKCα with WT mice in normal circumstance, while significantly increases PKCα activity when treated with CS for 3 months (Figure 6C). The qPCR results also supported that expression levels of ADORA2B and CD73 significantly increased in the CS group when comparing to Air group (Figure 6D). Lung pathology revealed that epithelial hyperplasia in bronchioles appeared to be present in WT mice after exposure to CS 3 months. The major adverse change was the presence of fibrosis-like lesions consisting of connective tissue components, extracellular matrix and collagen. CD73 or ADORA2B knockout mice have showed some improvement, and CD73/ADORA2B DKO mice showed remarkable improvement as compared to the CS-treated WT mice (Figure 6E). Picro-Sirius Red staining of lung sections to detect collagen deposition (red color in Figure 6F) showed more abundant collagen accumulated at the sites surrounding bronchioles in CS-treated WT mice while comparing to control group, the CD73/ADORA2B DKO mice significantly reduced the collagen deposition than ADORA2B KO or CD73 KO group (Figure 6G). Together, these results demonstrate that double knockout CD73 and ADORA2B remarkably improved CS-induced lung injury by activating PKCα, reducing the inflammatory cell number in BALF, inhibiting the fibrosis-like lesions and decreasing collagen deposition surrounding bronchioles.

## Discussion

**FIGURE 7:**
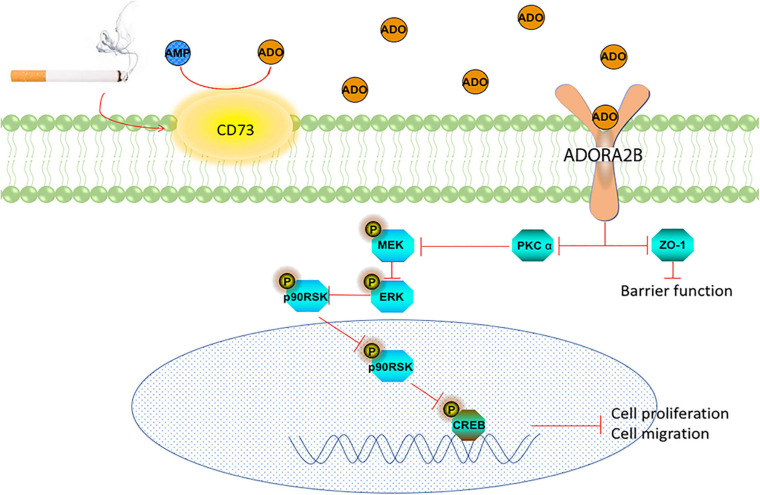
Schematic representation of the role of CD73 and ADORA2B in CS impairment of wound repair in airway epithelial cells.

The epithelium provides the airway a barrier against inhaled environment toxins and airborne pathogens and can initiate a variety of responses when injured, such as rapidly supporting repair processes. Extracellular accumulation of ADO in response to tissue damage is an important indicator for control of wound repair. ADO has been known to have both anti-inflammatory (tissue-protective) and pro-inflammatory (tissue-destructive) properties (Hasko and Cronstein, 2013). The nature of ADO’s action depends on the magnitude of changes in extracellular concentrations and the expression levels of each AR subtype. We previously reported that ADO promotes wound repair through ADORA2A signaling pathway in acute injury in bronchial epithelial cells (Allen-Gipson et al., 2006, 2007). Recently, we demonstrated that CS exposure increases ADORA2B expression level in mice (Tian et al., 2017). This study was designed to understand the mechanisms of ADO shifting from its tissue-protective to tissue-destructive features in long-term CS exposure. We found that long-term CS exposure upregulates the transcriptional and protein expression of CD73 and ADORA2B, increases ADO production and decreases PKC alpha activity. This was a marked contrast to our earlier findings where PKC alpha was increased in our 6 weeks *in vivo* model (Tian et al., 2017). Furthermore, our findings revealed long-term CSE inhibits the ERK signaling pathway resulting in the dysregulation of airway wound repair (Figure 7).

Airway epithelial cells act differently in different stages of CS exposure. Rubio et al. (2017) reported that CS exposure increases proliferation and migration in epithelial cells28, and other groups demonstrated progressive morphological changes and epithelial-to-mesenchymal (EMT)-like phenotype in normal airway epithelial cell after CS treatment (Wang et al., 2015; Vaz et al., 2017). We also observed that CSE exposure increases proliferation and migration, and EMT-like phenotype in the early stage (data not shown); however, long-term CSE exposure significantly impaired cell proliferation, migration and tight junction integrity after 3 years exposure, consistent with Das’s observation that CSE exposure disrupted cell structure and tubulin-microtubule function in lung epithelium cells (Das et al., 2009). Our microarray analysis indicated that long-term CSE exposure upregulated matrix metalloproteinase-1 (MMP1) expression and downregulated Col5A1, Col5A2, and Col6A1 gene expression. These findings suggest CSE plays a tissue destructive role in long-term exposure while it promotes cell activity in the early stage of wound repair.

Mitogen-activated protein kinases (MAPKs), including extracellular-signal-regulated protein kinase (ERK), stress-activated protein kinases (SAPK) p38 and SAPK c-jun N-terminus kinase (JNK), are well known to play roles in barrier function, proliferation and migration (Olson et al., 2009; Crosby et al., 2011; Mihai et al., 2012); ADORA2B can affect proliferation and migration through all three pathways (Kuno et al., 2008; Darashchonak et al., 2014; Merighi et al., 2017); however, there is still no research that focuses on how ADORA2Bs regulate the MAPK pathway in airway epithelial cells in wound repair. While several studies have indicated that ADORA2B plays a tissue-protective role in acute lung injury, it may become tissue-destructive in chronic lung injury (Karmouty-Quintana et al., 2012; Hoegl et al., 2015). Sun et al. (2006) reported that chronic lung disease in ADA-deficient mice is partially mediated by ADORA2B10. Other researchers also demonstrated ADORA2B’s highly distinct roles in acute and chronic stages of bleomycin-induced lung injury (Zhou et al., 2011). However, the exact mechanisms involved remain unclear. PKC alpha is well known to promote the phosphorylation of ERK signal (Ueda et al., 1996; Clark et al., 2004; Giacomelli et al., 2018). It is well documented that CS activates PKC alpha, including our previous study (Simet et al., 2010; Tian et al., 2017). However, we observed a decrease in the PKC alpha activity after 3 years CSE treatment in this study. The effects of CS on signaling may depend on the length of exposure of the cells and the concentration of nicotine or other constituents in the CS (Mehta et al., 2008). PKC alpha has been reported to promote cell proliferation and migration (Wu et al., 2008; Gao et al., 2009). In our long-term CSE exposure cell model LTC, cell proliferation and migration have been significantly decreased, which is consistent with the decreasing of PKC alpha activity.

We have also investigated whether targeting ADORA2B or upstream CD73 could improve long-term CSE impaired airway epithelial cell wound repair. Our data suggest that knocking down ADORA2B and/or CD73 could significantly ameliorate airway epithelial cell wound repair via activating PKC alpha/MEK/ERK/p90RSK/CREB pathway. Interestingly, rather than displaying redundancy, knocking down both ADORA2B and CD73 revealed to be more potent than knocking down either alone. Increasing evidence indicates that ADORA2B is a potential therapeutic target in not only lung injury (Huerter et al., 2016; Philip et al., 2017) but also in other diseases (Molck et al., 2016; Alencar et al., 2017; Ballesteros-Yanez et al., 2017; Borea et al., 2018). In this study, we demonstrate that knocking down ADORA2B increased CD73 expression in LTC, which indicates that the lack of ADORA2B creates a negative feedback loop via CD73 upregulation, in its attempt to increase ADO level and subsequently to activate ADORA2B. Similarly, Young et al. (2016) also observed that CD73 expression level was increased in the tumor core of ADORA2A deficient mice54. As a proof of concept for the synergistic interaction of the combination with CD73 and ADORA2B inhibition, the increasing of CD73 caused by ADORA2B inhibition was also decreased when co-inhibiting both CD73 and ADORA2B. Especially, the CD73/ADORA2B DKO mice demonstrated dramatically decreasing in the production of inflammatory mediator IL-6 and collagen deposition surrounding bronchioles than knock out CD73 or ADORA2B alone. This provides an important insight for understanding AR(s) drug targeting, especially when utilizing specific AR antagonist(s) which may promote a high level of CD73-generated ADO. It has been well documented that prolonged increases in ADO levels can promote pulmonary inflammation airway remodeling, and causes pulmonary tissue destruction (Zhou et al., 2009). *In vivo* experiments also confirmed that double knockout CD73 and ADORA2B significantly improves CS-induced lung injury. Collectively, co-targeting AR(s) and upstream CD73 has more therapeutic potential than targeting either individually.

In conclusion, Figure 7 summarizes our proposed role of ADORA2B on long-term CSE exposure induced wound repair. During long-term exposure, CSE induced overexpression of CD73 increasing ADO production, which subsequently activates ADORA2B. The activation of ADORA2B stimulates PKC alpha activation and inhibits MEK/ERK/p90RSK/CREB signaling pathway, which eventually inhibits airway wound repair. Moreover, co-targeting ADORA2B and CD73 exhibited to be more potent than targeting either individually in improving CS related airway wound repair.

## Data Availability Statement

The datasets presented in this study can be found in online repositories. The names of the repository/repositories and accession number(s) can be found in the article/supplementary material.

## Ethics Statement

The animal study was reviewed and approved by the USF Institutional Animal Care and Use Committees.

## Author Contributions

ZT, JD, BD, and XG performed experiments presented in the manuscript. FC analyzed microarray data. ZT and DA-G designed experiments presented in the manuscript. ZT, JD, QL, YZ, and DA-G prepared and approved manuscript for submission. All authors contributed to the article and approved the submitted version.

## Conflict of Interest

The authors declare that the research was conducted in the absence of any commercial or financial relationships that could be construed as a potential conflict of interest.
